# What Do We Know About Children’s and Adolescents’ Formal and Non-Formal Learning in the Zoo? A Systematic Literature Review

**DOI:** 10.3390/ani15243533

**Published:** 2025-12-08

**Authors:** Armin Baur

**Affiliations:** Department of Biology Education, Institute for Science and Technology Education, University of Education Heidelberg, 69120 Heidelberg, Germany; baur@ph-heidelberg.de

**Keywords:** zoo, aquarium, learning, literature review, children, adolescents

## Abstract

Zoos and aquariums are important learning environments for formal, non-formal and informal learning. A systematic theoretical basis for adult visitors’ learning at zoos and aquariums already exists. In total, there have been six literature reviews of this topic. However, to date, no literature review has been published concerning children’s and adolescents’ learning in zoos and aquariums. The systematic literature review presented in this article fills this gap and summarises the published results on formal and non-formal learning by children and adolescents in zoos and aquariums. A literature search in the Education Resources Information Center (ERIC) and Web of Science databases yielded 858 peer-reviewed articles. Fifty-one of these met the inclusion and exclusion criteria and were analysed more deeply. Each step of the literature search was verified by a human interrater. The analysis of the studies included in the review followed the steps of qualitative content analysis. The coding of the studies was done by two raters to ensure objectivity and reliability. For these, a human rater and an artificial intelligence system analysed the studies. The results indicate differences in research focus in different countries and that zoos and aquariums are productive learning environments for various types of visiting (e.g., learning trips, family outings) with a variety of identified learning methods and elements (e.g., touch tanks, keeper talks, guided tours, camps) that have different positive effects on knowledge, attitudes, behaviour and other variables. However, the results also identify starting points for new research and research gaps in this area.

## Simple Summary

While adult learning in zoos and aquariums has been systematically reviewed, no previous literature review has focused on children’s and adolescents’ learning. This article fills that gap by presenting a systematic review of studies on formal and non-formal learning by young visitors in zoos and aquariums. A search in two databases identified 858 peer-reviewed articles, of which 51 met inclusion criteria and were analysed in detail. The selected studies were examined through qualitative content analysis and a mixed-methods approach. The findings reveal international differences in research focus and confirm that zoos and aquariums provide effective learning environments for various visit types, such as learning trips, family outings and camps. Learning methods including touch tanks, keeper talks and guided tours were found to positively influence knowledge, attitudes and behaviour. The review also highlights current research gaps and suggests directions for future studies.

## 1. Introduction

Zoos and aquariums are places of learning that offer a wide range of activities for schools, kindergartens, families and individual visitors, and they are very popular. The term “zoo” is a collective term for zoos, zoological gardens or wildlife parks. The terms “zoological garden” and “wildlife park” are often used for zoos that span a large area [1]. Some zoos include aquariums, but there are also stand-alone aquariums. In 2018, German zoos offered 171,426 special educational programmes, which were attended by 1,243,948 visitors [2]. But what do we know about the resulting learning at the zoo with regard to non-adult visitors? The following first describes zoos and aquariums as places of learning and summarises what is currently known about educational programmes in zoos and aquariums. This information is then used to derive the questions and objectives of this literature review, which compiles and analyses the current state of knowledge in order to provide teachers and zoo staff with learning methods and elements for educational programmes and to highlight for scientists the research needs for further studies.

### 1.1. Zoos as Educational Institutions

Historically, zoos and aquariums were menageries, where wild animals were displayed to a wealthy and aristocratic audience. By contrast, today zoos and aquariums are places of education for a broad audience. Education—especially education for sustainable development—has become one of the focuses of the work of zoos and aquariums around the world [3]. Learning can take place in various locations outside of schools: these are referred to as extracurricular learning locations [4]. One such extracurricular learning location is the zoo or the aquarium (both of which are grouped below under the term “zoo” for ease of reading). The zoo as a place of learning should not be underestimated as animals are an important part of children’s lives [5,6]. Zoos also provide real impressions of animals, thus opening up learning processes. For a long time, it was widely believed that learning at school was the key for children and young people to acquire new knowledge. In recent years, however, it has become clearer that out-of-school institutions also make an important contribution in this regard [7,8]. Learning takes place not only at school and in the classroom. If extracurricular locations are to be used as opportunities for formal learning, a methodical three-step approach is recommended: first, preparation of the learning; second, learning at the extracurricular location; and third, follow-up of the learning [9,10]. But what other aspects for formal learning of students in the zoo need to be considered? And what is the case for non-formal learning by children and adolescents? Learning that is non-formal and, therefore, often unexpected, cannot be easily investigated using research methods developed for formal learning events [11]. Thus, we know more about formal learning and less about non-formal learning.

### 1.2. Existing Literature Reviews on the Topic of Learning in the Zoo

Despite the limited availability of investigations into non-formal learning, there are many literature reviews about general learning in the zoo. Five literature reviews and one meta-analysis have previously been published about learning in the zoo (literature reviews see Refs. [12,13,14,15,16]; meta-analysis see Ref. [17]). To make this manuscript easier to read, the term “literature review” will be used below for the five review articles and the meta-analysis (this was also done in the abstract for calculating the number of reviews). The following sections compare the six literature reviews and present their objectives, results and discrepancies (for a systematic comparison in the form of a table, see Table A1 in Appendix A).

#### 1.2.1. Focuses of the Literature Reviews

Comparing the six publications reveals that five of them focus on nature conservation education only. Other variables commonly examined when studying scientific education (e.g., knowledge, beliefs and motivation) which do not belong primarily to conservation education (e.g., memories of experiences at a zoo summer camp [18]; anthropomorphic thinking [19]; interest in science [20]) or about fostering other competencies which are not directly related to science education (e.g., performance in a vocabulary test [21]) are ignored in these reviews. However, some of these variables could also have an indirect or hidden impact on conservation awareness, while some relate to education more generally and should remain subject to research about zoos’ educational effects. Indeed, Xi and colleagues [22] found that there is a correlation between attitudes to science and environmental awareness, while Härtel and colleagues [23] report that students’ species knowledge affects both their environmental systems knowledge and their attitudes toward the environment.

#### 1.2.2. Differentiation According to the Age of the Subjects in the Studies of the Literature Reviews

In five of the six literature reviews, the subjects in the samples of the included studies are only zoo visitors in general (mostly adults). The only publication that differentiates between younger persons and adults is that of Dierking and colleagues from 2002 [16]. This article is now over 20 years old and differentiates children and adolescents from adults only in partial aspects. Thus, there is a gap to be filled in the comparison of existing studies about learning in the zoo. Indeed, the learning of adults and non-adults differ in various points. For example:

- Learning activities for children and adolescents in the zoo are often a part of activities organised by schools, so they are often not a free-choice learning; by contrast, learning activities for adults are rarely formal and nearly always “free-choice learning” (see Ref. [24]).
- The cognitive capacities of adults and non-adults are different (see, e.g., Jean Piaget’s theory of cognitive development, Ref. [25]).
- Another difference between adults and children or young people are their prior experiences. Adults have generally gathered more experiences given the longer time they have lived [26]. Prior experiences build up concepts that have an essential effect on learning (see, e.g., conceptual change theory [27]).

The differences between adults and non-adults make it necessary to build on existing research with a focus on children and adolescents and to complement the existing literature reviews.

#### 1.2.3. Learning Arrangements Investigated by the Studies in the Literature Reviews

This section summarises the teaching and learning methods, media and characteristics of the learning environment that are investigated in the studies as “learning arrangements”. Learning arrangements listed in the reviews include digital media, videos, touch tables, design of the enclosure/aquarium/terrarium, teaching and learning sequences, zoo visits, walkways, guided tours, conversations with animal keepers, interactions with live animals, fact cards, signage and zoo programmes (e.g., Refs. [14,17]). In the review by Mellish and colleagues [14], frequencies of the investigations are presented. The most commonly examined arrangement is the visit to the zoo (24.5%), followed by the speech or keeper talk (17.0%).

#### 1.2.4. Operationalisation of the Effects in the Studies in the Literature Reviews

Given their focal points, many of the literature reviews include studies that measure dependent variables related to conservation education. These are mostly knowledge, beliefs, behaviours and intentions [12,13,15,16,17], but studies that measure perception [13] are also included. One review [14] does not provide any information on the dependent variables. Another highlights the diversity and inconsistency of the terms of variables used in the studies [15].

#### 1.2.5. Research Methods of the Studies in the Literature Reviews

In the studies in question, the following research methods were used: questionnaires, interviews, observations, games and card laying methods [14,15,16]. Godinez and Fernandez [13] found in their analysis that a control group is often missing in the research designs of the studies. According to these results, there is a lack of research comparing zoo visitors with non-visitors to determine the actual impact of zoos.

#### 1.2.6. Results of the Studies in the Literature Reviews

All six works show effects of different studies that surveyed various learning arrangements. According to the meta-analysis by McNally and colleagues [17], a keeper talk, a general zoo visit, educational programmes and interactions with living animals have an positive effect on different dependent variables. A keeper talk has a moderate effect on knowledge (Cohen’s d = 0.65) and a small effect on intention (Cohen’s d = 0.25) and attitude (Cohen’s d = 0.17). A general zoo visit has a moderate effect (Cohen‘s d = 0.56) on knowledge and a small effect on attitude (Cohen’s d = 0.21). Interaction with living animals leads to small effects on the variables of knowledge (Cohen’s d = 0.34), intention (Cohen’s d = 0.24) and attitude (Cohen’s d = 0.38). Formal education (zoo education programmes) has a moderate effect on knowledge (Cohen’s d = 0.60) and attitude (Cohen’s d = 0.60). Also, other literature reviews show that zoos as educational institutions have a positive impact on the conservation knowledge, attitudes and behaviours of visitors [13,16]. Repeat zoo visits are very productive, according to Godinez and Fernandez [13], because more frequent visitors show significantly more conservation knowledge, have more strongly positive attitudes toward nature and are more involved in conservation activities. These results show that while visitors do not come to zoos to learn more about nature conservation, they are nonetheless open to learn more about it when viewing animals [16]. Meanwhile, teaching and learning materials that are especially interactive, experience-oriented and emotion-provoking have positive effects [16]. Furthermore, zoo visitors are often already very conscious of nature conservation and see animals as very worthy of protection [15]. Finally, in addition to the learning environment, the social context and the learning material are very important [15].

### 1.3. Objectives and Research Questions

As is clear from the six literature reviews, numerous studies on learning processes at zoos have been published in recent years. These studies report on formal, non-formal and informal learning arrangements that vary in terms of methodology and content and that have a wide range of effects in various dimensions. However, these literature reviews pay little attention to children and young people. There is no review that explicitly focuses on this. Children as a group of zoo visitors have to date been relatively little studied [28,29]. Future research should look into them more: this is one objective of the present work. Another objective is derived from some of the literature reviews not being detailed in all aspects of their data collection. For example, two literature reviews did not provide any information on the time span of the studies they included. This contradicts a newer understanding of literature reviews, known as “systematic literature reviews”:

*“In contrast to traditional or narrative literature reviews, that are criticized as being biased and arbitrary, the aim of a systematic review is to carry out a review that is rigorous and transparent in each step of the review process, to make it reproducible and updateable”* [30].

According to the requirements described—namely, a literature review about the learning of children and adolescents in the zoo, other variables not included in the six reviews and the importance of systematic literature reviews—the following research questions were established:

- (RQ1) What teaching and learning methods are used for teaching and learning processes at the zoo with school classes/adolescents/children?
- (RQ2) How are the effects on teaching and learning processes or the learning environment in relation to school classes/adolescents/children at the zoo operationalised in the published works?
- (RQ3) How are these effects measured using research methods?
- (RQ4) What effects on school classes/adolescents/children have been identified so far, and how are these related to teaching and learning methods or the learning environment?

Answering these research questions will lead to directions, ideas and help for creating and developing learning arrangements in zoos to improve environmental conservation knowledge, attitudes and behaviours. The answers will also shed light on other aspects which could be addressed by zoos to improve environmental conservation variables. Furthermore, this review will highlight possible research gaps and methods for further research in the field of zoos as educational institutions.

## 2. Materials and Methods

The process of searching for and analysing the literature was split into four steps: (1) search for literature, (2) selection of studies to be included, (3) qualitative analysis and creation of a data matrix, and (4) data analysis using the data matrix.

### 2.1. Search for Literature

In accordance with the recommendation by Siddaway and colleagues [31], two databases were used to identify relevant studies. The two databases were Web of Science and the Education Resources Information Center (ERIC). These were selected because the ERIC database contains a large number of works from the field of education (subject-specific and educational journals and books), and the Web of Science database covers a wide range of scientific journals that are not only subject-specific and educational. The search focused on published works that were peer reviewed and contained specified search terms (see Table 1). The search terms were determined using a rating procedure and open hints by a group of experts (researchers in biology education and zoo animal biology who are actively involved in research on this topic). The following individuals were involved: Prof. Dr. Annette Scheersoi (biology education, University of Bonn), Prof. Dr. Matthias Wilde (biology education, University of Bielefeld) and Prof. Dr. Paul Dierkes (zoo animal biology, University of Frankfurt). 

Only English-language articles were included. The following attributes were also set in the search filters in the databases:

(1) Publications are peer reviewed: This applies to all publications included in the Web of Science database: “The peer review status of a journal is a requirement in the journal evaluation process for inclusion in Web of Science Core Collection” [32]. The use of peer-reviewed publications should maintain the quality of the included works at a reliable level.
(2) Publications are articles from journals: This usually goes hand-in-hand with the attribute of being peer reviewed.
(3) Publications from the years 2000–2024: This allowed 25 years to be covered in the literature review.

If the number of hits for a search term combination exceeded 350, the search was narrowed down using “AND (Student* OR Pupil* OR Child*)”. This was done because, although the search terms were intended to cover as much content as possible in relation to the objective of the literature review, the amount of literature to be reviewed had to remain manageable [33].

A total of 1198 publications were found using the term combinations and filter settings. After duplicates were deleted using the reference management software Citavi version 6.14.0.0 [34], there were 858 publications.

### 2.2. Selection of Studies to Be Included

This step involved the two sub-steps (A) title and abstract screening and (B) full-text screening. In the first sub-step, the titles and abstracts of the publications found were checked against defined inclusion and exclusion criteria (see Table 2). In the second sub-step, the same was done with the full text of the articles and the same criteria. The selection of articles was checked for reliability and objectivity for each of the two sub-steps using an interrater and the determination of Kappa (κ) and percentage agreement (PA). A research assistant was involved as the interrater to perform the abstract, title and full-text screenings on all sources. For the organisation of the inclusion and exclusion criteria, the PI/EMS (population, intervention/exposure, measure, study design) framework of Hempel [35] was used.

If a study included a mixture of adults and non-adults in the population, an essential inclusion criterion was that the results of the non-adult sub-sample could be clearly separated from the overall results. In parallel, this literature review analysed learning in zoos/aquariums. For this reason, only studies that examined this topic were included. Studies of learning experiences that were offered by zoos or aquariums but did not take place in them, such as the study by Fortescue and colleagues [37], which examined learning experiences in local waters, were not included. Studies conducted in national parks or nature conservation centres were also not included. The focus of this literature review is on zoos and aquariums, in other words pre-structured learning environments where animals are kept in enclosures, terrariums or aquariums. Studies that took place at zoos/aquariums, but did not explicitly examine these locations as places of learning, such as the study by Lawrence and colleagues [38], were also not included. In that study, zoos were examined as a distraction factor for students with attention deficit hyperactivity disorder and not as places of learning.

The interrater agreement for (A) title and abstract screening and (B) full-text screening was evaluated using the statistical software package R version 4.4.3 [39]. Interrater agreement showed very good agreement for both steps: abstract and title screening: κ = 0.91, PA = 98% and full-text screening: κ = 0.91, PA = 96%. All discrepancies were discussed between the research assistant and the author for both the abstract and title screening and the full-text screening. The totality of the studies included in the review therefore reflected the author’s selection, taking into account the changes triggered by the discussions with the interrater. The interrater’s selection, taking into account the changes triggered by the discussions, was used to determine the interrater agreement coefficients κ and PA.

**Figure 1:**
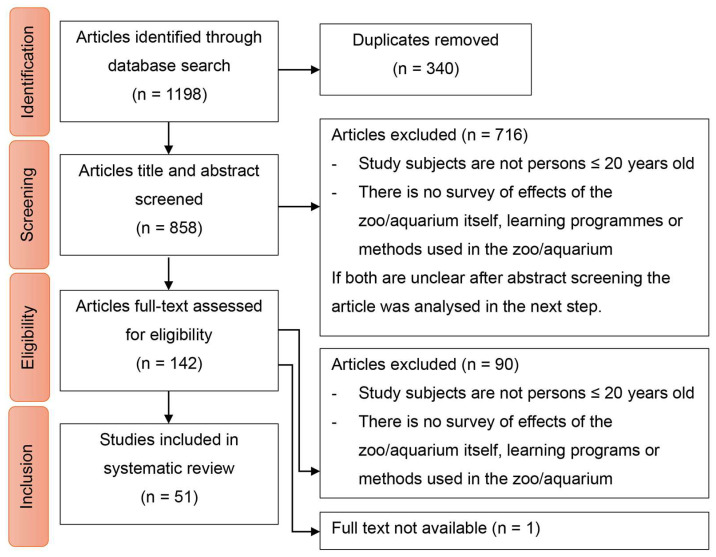
Flow chart illustrating literature search. Beside the names of each step of the search and selection process (red boxes), left column gives number of studies found or remaining after each process. Right column indicates number of studies excluded at each step and exclusion criterion.

In total, the title and abstract screening resulted in 142 studies being analysed in the full-text screening. The full-text screening led to 51 studies being included in step 3, “qualitative analysis and creation of a data matrix” (see Figure 1).

### 2.3. Qualitative Analysis and Creation of a Data Matrix

The studies remaining after the full-text screening were analysed using qualitative content analysis (open coding and category formation). A category system was developed and applied inductively (category system available from the author). The proposed steps for qualitative data analysis according to Kuckartz and colleagues [40] were used:

- Data exploration: Key points regarding the study design of the included studies were noted and a summary for each study was prepared, to obtain an overview when comparing studies and developing codes.
- Creation and application of the category system: The category system was created inductively from the data material and summaries, and definitions of the categories were formulated.
- Coding based on categories and describing results: The step was done in the original texts (studies) using the software MAXQDA version 24.8.0 [41], and the codes from each study were transferred to a data matrix in nominal scales; text sections and a summary of the result of each study were also transferred in the data matrix.
- Analysis: See Section 2.4.
- Conclusion drawing and consequence identification: See Results (Section 3).

The definitions of the categories were also transferred to prompts (prompts available from the author). The prompts were used in artificial intelligence (AI)-supported interrater work. In this step, there was a difference in the determination of the interrater agreement for the abstract, title and full-text screenings. The abstract, title and full-text screenings were performed by two human raters. In this step, however, the assignment of the inductively generated categories was performed by one human rater (author) and one AI system (AI-Assist from the software MAXQDA version Analytics Pro 24.8.0 [41]). The decision to use an AI system was made for technical and administrative reasons. First, the incorporation of new technologies appears to be an appealing option given the growing interest in AI-supported systematic reviews. Meanwhile, no additional research assistant was found who could act as a second rater. However, other studies have shown that AI can rate as well as humans. Therefore, this is not a limitation (e.g., Ref. [42]).

The percentage agreement was used as the parameter for the learning arrangements examined by the studies. Kappa values and PA were calculated for all other categorisations. As explained above, learning arrangements are elements and methods examined as independent variables in causal studies or as supervised variables in correlative and descriptive studies. The number of learning arrangements was so high (25 elements/methods found) that categorisation would have resulted in a very large category system, with categories often being assigned only once (this was the case for 15 of the 25 elements/methods). The respective κ and PA can be found in the results presented in Section 3.

### 2.4. Data Analysis Using the Data Matrix

In the final analysis step, a mixed-methods approach was used to make qualitative, descriptive and inference statistic statements. For this reason, a sequential design was used. In this sequential design, the data were first analysed qualitatively (bringing the categories into a data matrix) and then quantitatively. Therefore, the qualitative analysis influences the quantitative analysis [43].

Qualitative statements of the studies were identified, frequencies of the categories were calculated and significances analysed. Fisher’s exact test was chosen for significance testing because it can be used for nominal scales when the conditions of the χ^2^ test are not met, which was the case in the analysis. In cases where the contingency tables were too large, the Monte Carlo simulation was used in Fisher’s exact test to estimate the *p*-value. It is important to note that the *p*-value determined by the Monte Carlo simulation is an estimate that changes slightly with each recalculation. The number of simulations to be performed for the estimation was set at 10,000.

## 3. Results

### 3.1. Descriptive Statistics of the Studies

**Figure 2:**
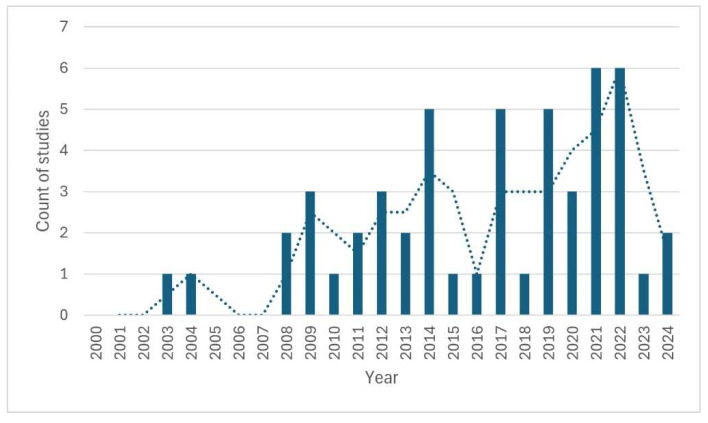
Publications by year. Dotted line shows moving average.

The studies found were published between 2003 and 2024. The diagram in Figure 2 shows that the number of publications gradually increased and repeatedly reached very high levels from 2014 onwards. The highest annual number of publications (six) on the topic of the literature review appeared in both 2021 and 2022. The largest proportion of studies were published by researchers from the United States (see Table 3). In some cases, researchers from different countries were involved, which leads to a different total number compared to the number of studies found. The variety of journals in which the studies were published was very large. Thirty-two different journals were identified in which the studies included in the final analysis step were published. The most common journals were *Environmental Education Research* (six studies), followed by *Science Education* (five studies) and *Frontiers in Psychology* (four studies).

**Figure 3:**
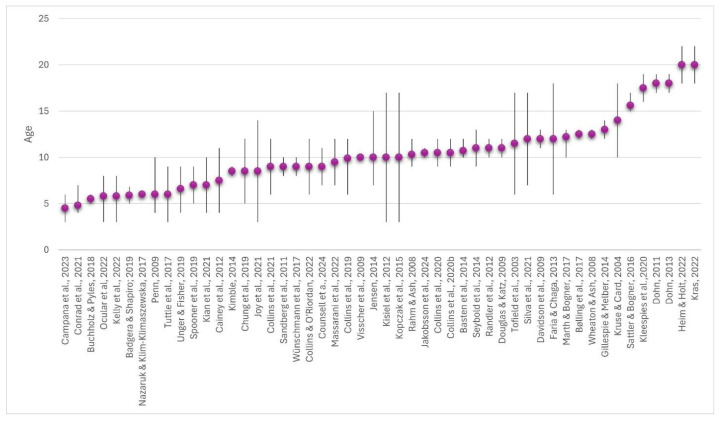
Overview of age distribution in samples of included studies: excluding study by Taylor and Duram [44], as it was retrospective. Dots show the mean and lines the range.

The sample sizes of the studies range from 2 to 2839 participants. The mean value is 293.6 participants. The average age of the samples in the reviews was between 4.5 and 20 years (see Figure 3), with the overall mean being 10.3 (SD = 3.74) years. As can be seen in Figure 3, many of the studies investigated children and adolescents aged about 10 years. It should be noted that some studies included both adults and non-adults as sub-samples. As explained above, only the sub-samples of non-adults were included in this literature review. One study [44] was retrospective in design, with adults reflecting on the impact of their previous visits to zoos in childhood and adolescence, and this was not included in Figure 3. In the descriptions of the study samples, it was noticeable that children and students with special educational needs were not examined or mentioned in any study on learning effects at the learning location.

**Figure 4:**
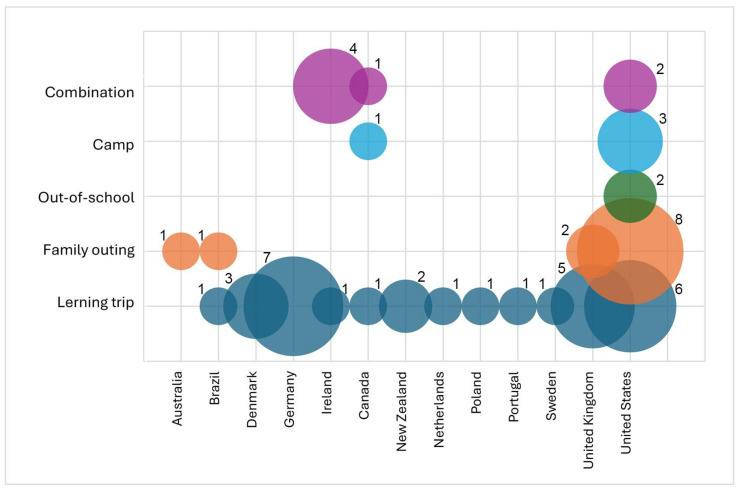
Type of visit to zoo/aquarium. Number beside each bubble indicates corresponding number of studies. Size of bubble corresponds to number: e.g., number of studies surveying learning trips is one in Brazil, five in the United Kingdom, six in the United States and seven in Germany; interrater agreement: κ = 0.85; PA = 90%.

The types of visits to learning locations (see Table 4, Figure 4) also differ between countries, but the differences are not significant (Fisher’s exact test with simulated *p*-value, p approximately 0.060). While all types are examined in the United States, many countries focus only on learning trips. Germany has the most studies of learning trips (seven studies). In the area of family outings, the United States is the most frequent country (eight studies). The types can be classified as formal and non-formal education (see Table 4). Overall, learning trips are predominant, with 56% of the studies (28 studies) examining them.

### 3.2. Learning Arrangements Investigated in the Studies Found (Results Related to Research Question 1)

A total of 25 learning arrangements were found. Of the 25 learning arrangements, 15 were examined in only one study each. During the analysis of the studies, the narrow understanding of RQ1 had to be broadened, as many studies did not examine teaching and learning methods, but rather other factors related to learning in the zoo. All the learning arrangements found are listed in Table 5. As explained in the section “Methods” for interrater agreement, only PA was used to verify this categorisation.

Among the studies found, investigations analysing causal relationships predominate (69% of studies), followed by descriptive analyses (25% of studies) and analyses of correlations (6% of studies). The interrater agreement of this categorisation is: κ = 0.80; PA = 90%.

### 3.3. Variables Measured in the Studies Found (Results Related to Research Question 2)

**Figure 5:**
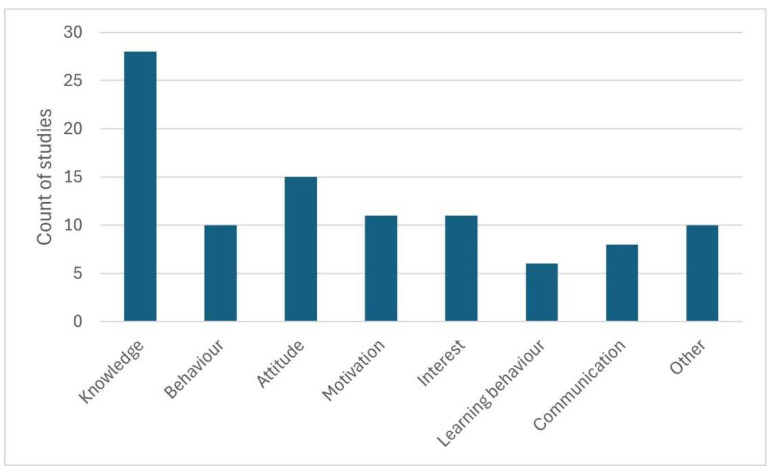
Frequencies of categories of dependent variable; interrater agreement: 0.62 ≤ κ ≤ 0.88; 82% ≤ PA ≤ 96%.

A total of seven frequently studied categories of dependent variables (see Figure 5 and Table 6) and ten dependent variables (category “Other”) that were only studied in a few studies were identified. Of the seven frequently studied variables, (changes in) knowledge was by far the most frequently studied variable. The effect on knowledge was studied in 28 studies (55%). The ten dependent variables that only appeared in individual studies were: memories (episodic memory, semantic memory) of experiences at a zoo summer camp [18]; anthropomorphic thinking [19]; performance in vocabulary tests [21]; smartphone use [45]; development of category-based induction in the biological field [46]; goals for zoo visits [47]; understanding of the topic [48]; culture [49]; ideas about science [50]; actions and interactions at the touch tank [51].

**Figure 6:**
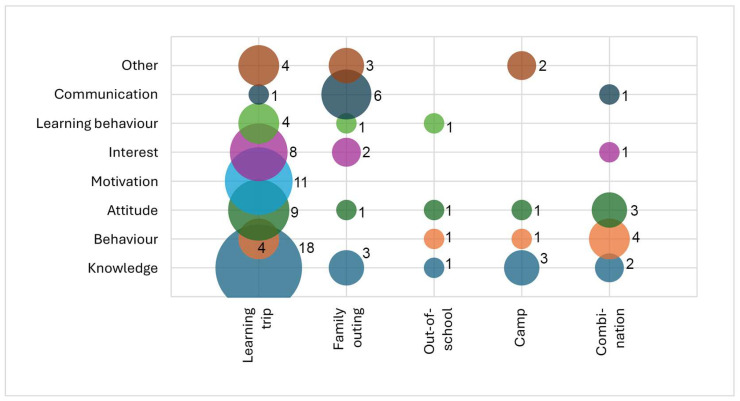
Frequency-dependent variables in relation to type of visit. Number beside each bubble indicates corresponding number of studies. Size of bubble corresponds to number: e.g., number of studies surveying knowledge is one for out-of-school setting, three for family outing and eighteen for learning trip.

The differences between the type of visit examined with regard to the inclusion of the dependent variables (see Figure 6) are not significant for the categories of knowledge (*p* = 0.201), attitude (*p* = 0.421), interest (*p* = 0.863) or learning behaviour (*p* = 0.506), but are significant for behaviour (*p* = 0.008), motivation (*p* = 0.022) and communication (*p* = 0.003). Behaviours are rarely examined in the learning trip (four of twenty-eight studies) and family outing (zero of ten studies) formats. Motivation is only examined in the learning trip (11 of 28 studies). Communication is not examined in the out-of-school setting and camp, almost never in learning trips (one study out of twenty-eight), but often in family outings (six out of ten studies).

### 3.4. Methodological Design of the Studies Found (Results Related to Research Question 3)

With regard to the didactic-methodological structure of the studies, it is striking that only 21% of the studies take the three-step methodological approach into account. In 61% of the studies, there is neither preparation nor follow-up. The interrater agreement of this categorisation is: κ = 0.72; PA = 84%.

With regard to data collection, 35% (18 studies) used three or four measurement points in their studies. Some 18% of the studies (nine studies) collected data at only one measurement point; of the studies that included only one measurement, two were descriptive in nature. Among the studies that examined causal relationships, only one study out of a total of 35 had four measurement points (3%), eleven studies used three measurement points (31%), sixteen studies had two measurement points (46%) and seven studies had a single measurement point (20%). The interrater agreement of this categorisation is: 0.84 ≤ κ ≤ 0.96; 92% ≤ PA ≤ 98%.

Analysis of the quality criterion included in the studies showed that 65% (33 studies) of all studies included in the analysis possess one or more of the following quality criteria: use of interrater; measurement of internal consistency of a test; use of a published test; piloting of a self-developed test; expert survey of the test during test development; use of published coding system. The interrater agreement of this categorisation is: κ = 0.78; PA = 90%.

The inclusion of a control group was not considered in 29 of the studies found (57%). If only the 35 studies that investigated causal relationships are taken into account, there are 13 studies (37%) that do not include a control group. The interrater agreement of this categorisation is: κ = 0.72; PA = 84%.

### 3.5. Survey Instruments Used in the Studies Found (Results Related to Research Question 3)

**Figure 7:**
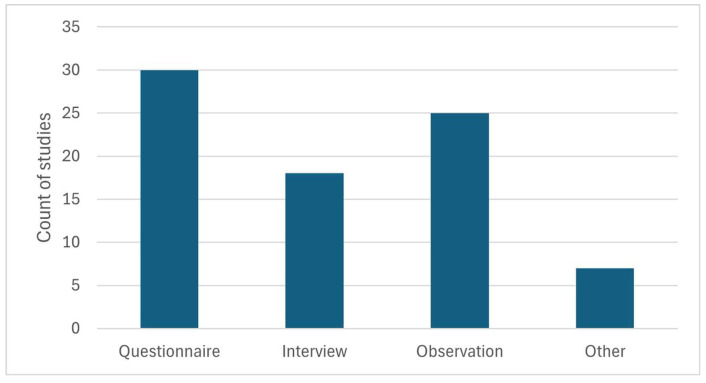
Frequency of survey instruments used (several different instruments could appear in each study); interrater agreement: 0.69 ≤ κ ≤ 0.96; 84% ≤ PA ≤ 98%.

The survey instruments used (see Figure 7) did not differ significantly in terms of the type of visit investigated (questionnaire: *p* = 0.146; interview: *p* = 0.767; observation: *p* = 0.175; other: *p* = 0.347).

However, there were significant differences in the survey instruments used in terms of the relationship investigated (causal relationships, correlation, descriptive study). Questionnaires were used significantly more often (*p* = 0.004) in the investigation of causal relationships and correlations. Interviews (*p* = 0.001) and observation (*p* = 0.000) were used significantly more often in descriptive studies.

### 3.6. Results of the Studies Found (Results Related to Research Question 4)

Many of the studies show positive effects of the learning arrangements examined (see also Table A2, Table A3, Table A4 and Table A5 in the appendix; interrater agreement of the effects of the studies included in the review: κ = 0.81; PA = 91%). The data collected in this review allow the following statements about the effects.

- The visit or learning trip to the zoo is reported (investigated in eight studies)—depending on the variables examined by the studies—as having an effective (positive) impact on knowledge, behaviour, attitudes, motivation, interest and learning behaviour (e.g., Refs. [52,53,54]).
- A lecture or guided tour has—depending on the results of the studies (examined in five studies)—a positive influence on the variables knowledge, attitudes, interest, motivation and learning behaviour (e.g., Refs. [29,55,56]).
- The educational programmes examined (examined in five studies) show—depending on the study—positive changes in knowledge, behaviour, attitudes and motivation (e.g., Refs. [57,58]).
- Depending on the study (investigated in five studies, some of which are related), the construction and use of enrichment has positive effects on knowledge, behaviour and communication (e.g., Refs. [28,59]).
- Direct contact with animals (examined in three studies) has a positive effect on attitude (e.g., Ref. [60]).
- Depending on the study (three studies examined this), the integration of a theatre play has a positive effect on the variables knowledge and learning behaviour (e.g., Refs. [61,62]).
- Parent–child conversations have a positive effect on attitudes, interest and communication itself, depending on the study (analysed in three studies; e.g., Ref. [63]).
- Attending a camp at the zoo has positive effects on the variable knowledge (examined in two studies; e.g., Ref. [64]).
- Virtual devices (examined in two studies) have an influence on knowledge and other variables (e.g., Ref. [45]).

In addition, individual studies found that:

- Active learning is also conducive to learning at the zoo (e.g., Ref. [55]).
- The animals at the zoo play an important/significant role in learning at the zoo (e.g., Ref. [65]).
- Conversations with zoo staff are stimulating (e.g., Ref. [56]).
- The sociodemographic variable of age and the provision of offers for people with a lower socioeconomic status have an effect on interest and knowledge (e.g., Refs. [18,20]).

However, some studies have also shown that the learning arrangements examined have no effect on the measured variables. There was no effect of whether the children came from rural or urban areas [66], or whether anthropomorphism was incorporated or not (this only had an effect on anthropomorphic attributions: children in the anthropomorphic condition attributed significantly more anthropomorphic characteristics to the animal involved than children in the realistic condition [19]), whether learning was self-directed or not [67] and whether there was a difference in the implementation of the programme at the zoo, at school, or both at the zoo and at school [68].

## 4. Discussion

### 4.1. Interpretation of the Results

The descriptive data show a trend that the annual number of studies published on children and young people’s learning at the zoo has increased in recent years; from one publication in 2003 to six in each of 2021 and 2022. Countries that are very active in this research field are located mainly on the continents North America, South America, Europe and Australia. The majority of studies conducted and published between 2000 and 2024 were carried out by researchers from the United States (twenty-one studies in total), followed by researchers from the United Kingdom (eight studies) and Germany (seven studies). This result may be influenced by the selection of English-language articles only, which must be taken into account. The focus on different types of visits (learning trip, family outing, out-of-school, camp) in the studies in the different countries is heterogeneous. However, the differences between countries are not significant. Researchers from the United States examined all types of visits. Researchers from Brazil, Ireland and Canada examined only two different types of visits. From Australia, only studies examining family outings were found. In the other countries, only the learning trip was examined. Overall, the learning trip is the most frequently studied type of visit (56% of studies). This shows that further research is needed on informal visits in different countries. 

The journals in which these studies have been published are numerous and come from different fields, such as journals focusing on science education, environmental education or psychology. The most frequent journal is *Environmental Education Research* (six studies), followed by *Science Education* (five studies) and *Frontiers in Psychology* (four studies). The differences in domains such as environmental education, psychology and science teaching make it very clear that learning at the zoo or the study of this can have very different objectives and that there is no publication organ specific to this topic.

It was also striking that none of the studies found explicitly examined learning effects in people with special needs. There is therefore a major deficit here, a need for further research. This group is not small and should not be forgotten. In 2020, 582,400 (statistically recorded) students with special educational needs were taught in special education institutions and mainstream schools in Germany, which corresponded to 7.7% of all students [69]. Since this group usually has different needs in terms of learning environments, this requires specific accompanying scientific research, some of which also has different focal points.

With reference to RQ1, “What teaching and learning methods are used for teaching and learning processes at the zoo with school classes/adolescents/children?”, numerous methods/elements (learning arrangements) were identified that were included in the studies found and examined (see Table 5 and the comparison in Table 7). The comparison (see Table 7) clearly shows that more learning arrangements were investigated in studies with children and adolescents than in studies with adults. This is certainly due to the fact that studies with children and adolescents concern formal and non-formal educational activities. Studies with adults can only analyse non-formal educational activities. The studies with adults also examined bus tours, tours and additional information material in forms of texts, all three of which are only suitable for school classes in exceptional cases. It would be interesting to ask zoos what different methods they use in practice. This could potentially identify further methods.

With regard to RQ2, “How are the effects on teaching and learning processes or the learning environment in relation to school classes/adolescents/children at the zoo operationalised in the published works?”, the following results were found (see Table 8). The studies involving children and young people mainly record the following seven dependent variables:

- knowledge
- behaviour (including intention)
- attitudes
- motivation
- interest
- learning behaviour
- communication

In adults, the variables knowledge, behaviour, intention and attitude were recorded [17,70]. In the studies evaluated in the course of this review, the variable communication was also found in studies with adults. Thus, far more different variables were used for children and adolescents. At this point, implications for future studies with adults could be derived, as further dependent variables such as motivation and interest could certainly be scientifically investigated in this field.

Evaluations of RQ3, “How are these effects measured using research methods?”, show that in 61% of the studies, there was no preparation nor follow-up to the learning experience at the zoo (31 studies). In 18% of cases, there was at least some preparation (nine studies). Only 21% involved both (11 studies). This approach contradicts the didactic-methodological recommendations of the three-step method. Furthermore, these three steps were not scientifically examined as a whole in any of the studies found. There was one study [68] that examined the variation of specific teaching in school and at the zoo with four groups (no specific teaching, specific teaching at school, specific teaching at the zoo, specific teaching at the zoo and school), where differences between the three groups with specific teaching and the group without specific teaching were found. However, the combination of the zoo and school programmes did not provide any additional benefit over the zoo programme alone. Unfortunately, the article does not specify an explicit order of the elements zoo and teaching for the combined zoo and school programme group; it merely mentions that this group received both forms of learning. In addition, it must be considered here, without criticism, whether the element examined really corresponds to preparation in the sense of the three-step method. Overall, there appears to be a great need for further research on preparation and follow-up. Although the three-step approach is recommended as important in the literature (cf. Refs. [9,10]), its importance in zoo settings has not yet been empirically proven or disproven due to a lack of studies. Looking at the research methodology of the studies, it is also striking that few of the studies use follow-up measurements. Only 35% (18 studies) of the analysed studies incorporate three or four measurement points in their research design. In 57% of the studies (29 studies), no control group was used. Scientific quality criteria were taken into account by 65% of the studies (33 studies). Overall, the methodological design of studies on learning effects in children and adolescents at zoos still has room for improvement. Three frequently used survey instruments/methods were consistently identified in the studies: questionnaire, interview and observation. These three types of surveys were used in qualitative and quantitative analyses. Several studies were also designed using a mixed-methods approach. The range of survey methods was correspondingly very broad and provides incentives for future studies.

In the analyses of RQ4, “What effects on school classes/young people/children have been observed to date and how do these relate to teaching and learning methods or the learning environment?”, teaching and learning methods and learning environments are understood to mean the methods and elements (learning arrangements) examined in the studies, which are listed in Table 5. Overall, the results of the studies included clearly show that the animals in the zoo play an important/significant role in learning at the zoo and that the learning environment is interesting and motivating, with touch tanks and direct contact with animals, for example, also enriching the experience. A lecture or guided tour has a positive effect on the knowledge, attitude, motivation, interest and learning behaviour of children and young people, but active learning is also conducive to learning at the zoo. The elements of theatre, virtual devices and enrichment construction were examined in several studies and show potential for supporting the learning processes that take place at the zoo. All these results clearly show that the zoo is a place where learning can take place in a methodically diverse and efficient manner. The sociodemographic variable of place of residence has no effect on the variables examined, whereas age and the provision of offers for people with a lower socioeconomic status has an effect on interest and knowledge. This suggests that, with regard to people with a lower socioeconomic status, school or community programmes offering visits to the zoo are helpful and should be supported. Individuals with lower socioeconomic status are less likely to visit the zoo with their families because of financial barriers to access.

Meanwhile, the use of anthropomorphism has no effect on knowledge acquisition. This shows that anthropomorphism does not support learning, but neither does it prevent it. Therefore, the use of this method is possible, but not essential. The educational learning programmes presented were also effective, but differed in their content and methodological structure. Both formal and non-formal educational offerings, such as a zoo camp or family outing are conducive to learning.

What answers do the results of RQ4 and the other RQs provide to the question asked in the title of this paper, namely “What do we know about children’s and adolescents’ formal and non-formal learning in the zoo?”

The studies included provide empirical evidence of effective learning programmes at the zoo, which also indicates that a great deal of intervention research is already being conducted at the zoo. The zoo as a place of learning is interesting and motivating for children and young people. Some methods/elements such as the construction and use of enrichment, animal contact, lectures and guided tours, theatre and virtual devices can support learning at the zoo. In addition, learning effects can also be empirically proven for non-formal offerings such as zoo camps and for informal learning through family outings with conversations between parents and children.

Overall, however, it can also be said that few elements/methods have been investigated and that informal and non-formal learning among children and young people has been researched little to date, depending on the country.

### 4.2. Limitations

As already mentioned above, only English-language studies were considered when selecting the articles included in this systematic literature review. As a result, there may be findings published in languages other than English that are not included in this review. An analysis of the countries in which the articles were published reveals a hotspot in English-speaking countries, which could also be an indicator that studies on learning in zoos are still largely published in the respective national languages. It is also striking that no studies from eastern and southern countries were found, which could also be an indication of bias due to the selection of English-language studies only.

It could also be seen as a limitation that additional search terms such as “educational excursion”, “educational trip”, “school trip” and “zoological garden” were not used to expand the search. Not using them may have resulted in a study not being included. However, as mentioned above, conducting such a review is a balancing act between the amount of data that can potentially be found and the feasibility of actually processing that data.

Another possible limitation is that studies are often only published if they show (significant) results. Studies that demonstrate that independent variables have no effect are rarely published [71,72]. This results in a publication bias, which may also be reflected in this literature review. This also becomes clear when looking at the effects in Table A2, Table A3, Table A4 and Table A5 in the appendix, which mainly contains studies with positive results.

Another limitation that should not be overlooked is the sometimes very superficial descriptions of the research methods and elements/methods (learning arrangements) examined in the included studies. These details were not always sufficiently detailed to provide a nuanced picture.

All of these limitations must be taken into account when interpreting the results presented above.

### 4.3. Implications

The results have the following implications for research:

- Research is needed on the impact and design of the three-step method (first, preparation of the learning, second, learning at the extracurricular location, and third, follow-up of the learning). None of the studies presented here explicitly examined the impact of the individual steps (as independent elements). In addition, there is a lack of research examining methods and design elements for the preparation and follow-up steps. Interesting questions for future research could include, for example: What effect/significance do preparation and follow-up have on the learning process in terms of cognition, motivation, interest, learning behaviour and environmental behaviour? How should preparation and/or follow-up be structured to best support the learning process? The method of repetition has already been examined in a study as an element at the end of a zoo visit, but not as a separate step in the follow-up, and has been shown to have an effect on interest and motivation [73]. Further analyses of methods such as reflection or task formats for consolidating and securing the content covered at the zoo are lacking.
- There is still a lack of further investigation of previously uninvestigated methods of teaching–learning work in zoos. These could be, for example, work on observation tasks, information boards, task formats or existing structural or functional models in the zoo. It would be very useful here to compare the methods that are already being used in practice and those that have already been investigated.
- There is a great need for research relating to children and young people with special educational needs. This research is lacking and must be implemented urgently.
- Another area with great potential for expanding learning at the zoo is informal and non-formal learning for children or young people. Thus far, research on this topic has only been conducted in Australia, Brazil, Canada, Ireland, the United States and the United Kingdom.
- In view of the significance of the results, further studies that include follow-up measurements are also needed.

The following conclusions can be drawn for educational institutions (schools, zoo schools):

- The zoo is a rich learning environment, and educational visits to the zoo are suitable for children and young people. Schools should therefore make use of this and include it in their planning. The informal and non-formal educational opportunities for children and young people are promising, according to the main findings of the studies, and should be further expanded.
- Many of the methods/elements (learning arrangements) presented and examined are suitable for practical use and can be implemented there.

Implications for education policy:

- The zoo is an important and interesting place of learning for children and young people and should therefore be used more extensively for formal education. However, this also requires educational policy and curricular implementations.

## 5. Conclusions

Research on learning in zoo environments is concentrated mainly in North America, South America, Europe and Australia. Most studies focus on learning trips, particularly involving children and adolescents around ten years old, while research on individuals with special needs remains lacking. The key dependent variables investigated are knowledge, behaviour, attitudes, motivation, interest, learning behaviour and communication. Although only a few studies applied follow-up methods, consistent findings highlight the educational value of zoo animals and the stimulating learning environment provided by zoos. Elements, such as touch tanks and direct animal contact, significantly enhance learning experiences. Both guided tours and lectures positively influence children’s knowledge, attitudes and motivation, while active learning further promotes engagement and retention. Innovative approaches, including theatre, virtual technologies and enrichment construction, show promising potential for supporting learning processes. While place of residence does not affect learning outcomes, age and socioeconomic factors influence interest and knowledge acquisition.

Research on zoo-based learning should further investigate the three-step method, diverse teaching methods, and the needs of children with special educational requirements, while also expanding studies on informal and non-formal learning and long-term effects.

Educational institutions and policy makers are encouraged to integrate zoos more systematically into formal learning, as many examined methods are practical and zoos offer rich learning environments for young people.

## Figures and Tables

**Table 1:** Search keyword combination. Boolean operators that were used are capitalised; these were AND and OR. Truncations () and phrase searches (“…”) were also used and are listed.

Search Keyword Combination	Justification
(1) Formal AND Learning AND (Zoo OR Aquarium)	High approval rating among experts: M = 4.83; SD = 0.37 (Max: 5–Min: 1).
(2) Learning AND Environment AND (Zoo OR Aquarium)	High to good approval rating among experts: M = 4.17; SD = 0.90 (Max: 5–Min: 1).
(3) Education AND (Zoo OR Aquarium)	High approval rating among experts: M = 4.83; SD = 0.37 (Max: 5–Min: 1).
(4) (“field trips” OR “field trip”) AND (Zoo OR Aquarium)	Note from the experts: Use “educational visit/trip” or “school trip”. A common term in the literature is “field trips”.
(5) informal AND learning AND (zoo OR aquarium)	Note from the experts: include “informal” or “non-formal”.
(6) (“out of school” OR out-of-school) AND (zoo OR aquarium)	Note from the experts: delete “setting” in “out of school setting”. (Agreement among experts with “setting”: M = 3.67; SD = 0.94 (Max: 5–Min: 1))

**Table 2:** PI/EMS framework.

PI/EMS	Inclusion Criteria	Exclusion Criteria
*Population* “The population criterion encourages you to characterize the study population that is included in the research you are looking for. A very broad characterization is ‘Are you looking for children or adults or both?’” [36]	Children and adolescents ≤ 20 years of age as well as adults who report retrospectively Target group: Kindergarten children, primary school children, secondary school children in grades 7–9 and 10–12 (in the United States, undergraduate students were also included)	Adults > 20 years of age who do not report retrospectively
*Independent variable or intervention/exposure* “The intervention is the independent variable in an experiment (the part you manipulate to find out its effects). It is the treatment the treatment group receives in intervention research studies. In the context of observational studies, this domain is the exposure of interest (e.g., classroom size when you are evaluating the effect of classroom size on school grades). In any other context this is the element that you think will have an effect on something or someone (e.g., personality traits when you are evaluating the effect of personality traits on college success).” [36]	Exposure of interest: learning environment: zoo or aquarium	Learning environment is neither a zoo nor an aquarium
*Measure* “This dimension should characterize the type of data you are interested in, that is, what the study should assess and report on to be of interest to you. This is about the type of assessment and the measure (e.g., test scores) that were used to determine whether there was an effect or what kind of effect there was. This is the dependent variable in experiments. In the biomedical literature this is the outcome or effect measure.” [36]	The effects of the learning location or effects related to them on visitors/learners were surveyed	No effects were recorded
*Study design* “This dimension focuses on the type of publication, research study, and methodological approach you are looking for. Are you interested only in empirical studies that report data, or theoretical papers as well? Can the study be a write-up of an experiment or an observational study? Must the publication report quantitative or qualitative data to be of interest? Does the paper need to report a particular analytic method (e.g., multivariate analysis)? And so forth.” [36]	Qualitative and quantitative data	No data were recorded

**Table 3:** Number of studies by country. Countries ascribed to each publication are those where the institutions where the respective author(s) work(s) are situated. Note: In some cases, articles with multiple authors have been assigned to multiple countries.

Country	Studies
Australia	1
Brazil	2
Canada	3
Denmark	3
Germany	7
Ireland	5
Netherlands	1
New Zealand	2
Poland	1
Portugal	1
Sweden	1
United Kingdom	8
United States	21

**Table 4:** Types of visits in studies.

Type of Education	Type of Visit to the Zoo/Aquarium
Formal education	*Learning trip:* Visitors visit the zoo or aquarium in connection with an event organised by their school, university or kindergarten.
Non-formal education	*Family outing:* Visitors visit the zoo or aquarium with their families. *Out-of-school:* Visitors do not visit the zoo or aquarium as part of a school, university, or kindergarten event, nor do they visit with their families. *Camp:* A camp is a special form of out-of-school setting. A camp lasts several days and includes overnight stays.
---	*Combination:* e.g., Taylor and Duram [44] collected data during a learning trip and family outings; Rahm and Ash [20] collected data during family outings and out-of-school programmes.

**Table 5:** List of learning arrangements investigated in studies found; in parentheses are shown counts of studies in each case: for those with no value given, value is 1; interrater agreement: PA = 94%.

Age Anthropomorphism Camp (2) Combination of school lessons and zoo Conversation with animal keepers (2) Direct contact with animals (3) Enrichment (5) Inclusion of virtual media (2) Learning trip (7) Lecture/guided tour (5) Motivation through accompanying persons Navigators Parent–child conversations (3)	Participation in animal care Participation of socially disadvantaged people Repetition Self-directed learning Specific educational program (5) Station work Student-centred approach Theatre (3) Touch tank Urban vs. rural children Zoo educator Zoo visits

**Table 6:** Categorisation and definition of dependent variables.

Category of Dependent Variable	Definition
Knowledge	Knowledge about animals, nature, the environment, species protection or nature conservation.
Behaviour	Reported or observed environmental behaviour, nature conservation actions or intentions to act.
Attitude	Attitude toward nature, animals or the environment.
Motivation	Self-reported or observed motivation in learning or in lessons.
Interest	Interest in science, animals, nature and/or the environment.
Learning behaviour	Observable behaviour in the learning process, participation in class, occurrence of disruptions and/or refusal to learn. Does not include communication between learners and family members.
Communication	Conversations with others.
Other	Unlike the variables described.

**Table 7:** Comparison of learning arrangements studied in adults and children/adolescents. X: learning arrangement is found, “---”: learning arrangement is not found.

	Children and Adolescents	Adults (McNally et al. [17]; Mellish et al. [14]; Information of the Studies in This Review)
Lecture/guided tour	X	X
Repetition	X	---
Station work	X	---
Self-directed learning	X	---
Inclusion of virtual media	X	X
Enrichment	X	---
Direct contact with animals	X	X
Conversation with animal keepers	X	X
Theatre	X	X
Anthropomorphism	X	---
Parents as navigators	X	---
Touch tank	X	X
Zoo educator	X	X
Camp	X	---
Participation in animal care	X	---
Additional texts	---	X
Tour of a zoo/aquarium	---	X
Bus tour	---	X

**Table 8:** Comparison of dependent variables investigated in adults and children/adolescents. X: learning arrangement is found, “---”: learning arrangement is not found.

	Children and Adolescents	Adults (McNally et al. [17]; Mellish et al. [14]; Information of the Studies in This Review)
Knowledge	X	X
Behaviour (also intention)	X	X
Attitude	X	X
Motivation	X	---
Interest	X	---
Learning behaviour	X	---
Communication	X	X
Others	X	X

## Data Availability

The raw data supporting the conclusions of this article will be made available by the authors on request.

## Appendix A

**Table A1:** Overview of previous reviews.

	McNally et al. [17]	Schilbert and Scheersoi [12]	Godinez and Fernandez [13]	Mellish et al. [14]	Nygren and Ojalammi [15]	Dierking et al. [16]
Thematic focus	Conservation education	Conservation education	Influence of the zoo on visitors	Conservation education	Conservation education	Conservation education
Groups of people involved	Zoo visitors in general (i.e., families, school classes, individual visitors)	Zoo visitors in general (i.e., families, school classes, individual visitors)	Zoo visitors in general (no differentiation in the text)	Zoo visitors in general (adults)	Zoo visitors in general (i.e., families, school classes, individual visitors)	Zoo visitors in general (adults and children)
Differentiation by age groups	No	No	No	No	No	In the areas of prior knowledge, attitudes, affect and behaviour, otherwise not
Selection criteria for the literature included	Interventions that were **only** offered by zoos At least one conservation-related variable measured	At least one conservation-related variable measured	Articles that investigated conservation awareness, attitudes and/or behaviour	At least one conservation-related variable measured	Unclear	Studies that measured the following:
						Prior knowledge, existing attitudes and previous environmental behaviour Effect of the visit to the zoo Adaptable methods
						or other relevant content
Time period (articles recorded from/to)	2017–2021	2011–2020	Not specified (maximum until 2019)	1998–2016	2007–2016	Not specified (maximum until 2002)
Operationalisation of effects of the studies (dependent variables)	Knowledge, beliefs, behaviour, intentions	Knowledge, beliefs, behaviour	Behaviour, perception, conservation efforts	---	Note that many of the studies use very different terms for their dependent variables	Conservation learning (knowledge, attitudes, affect, behaviour), perception of the animals
Research methods of the studies presented	---	---	There is no explicit statement on this except that a control group is often missing	Questionnaires, interviews, observations, games	Questionnaires, self-reports in conjunction with quantitative methods	Interviews, observations, questionnaires, Card-reading methods
Effects found	A visit to the zoo and interaction in the zoo have a positive effect on many variables	---	Zoo visits have a positive influence on visitors’ knowledge of nature conservation, attitudes and behaviour. Repeated visits to the zoo are particularly beneficial, as regular visitors have more knowledge about nature conservation, more positive attitudes toward nature conservation and are more involved in nature conservation activities	---	Visitors are usually already aware of nature conservation. The social context and the material after the visit support the learning objectives of the visit	Visitors do not come to zoos and aquariums specifically to learn more about conservation issues. Overall, the study results indicate that interactive, experience-oriented and emotionally appealing teaching/learning methods have positive effects

**Table A2:** Overview of studies found—Part 1. +: positive result, -: negative result, 0: no result, NA: not applicable. If ‘+’, ‘-’, ‘0’ or ‘NA’ appears, this was investigated in the study. If ‘X’ appears, this was used in the study.

No.	Article (Authors)	Year	Country of Authors	Journal	Type of Visit	Instrument				Time of measurement				Distance Follow-Up (Weeks)	Category of the Examined/Varied Variable	Investigated Relationship	Dependent or Observed Variable								Sample		
						Questionnaire	Interview	Observation	Other	Pre	On-Site	Post	Follow-Up				Knowledge	Behaviour	Attitudes	Motivation	Interest	Learning Behav.	Communication	Other	Age Min	Age Max	Sample Size
1	Taylor and Duram [44]	2021	USA	*Sustainability*	Family outing, Learning trip	X									Zoo visits	Correlation		+	+						18	68	136
2	Spooner et al. [61]	2019	Australia, UK	*Environmental Education Research*	Family outing	X				X		X			Theatre	Causal relationship	+								5	9	215
3	Kimble [52]	2014	UK	*Studies in Educational Evaluation*	Learning trip	X	X	X		X		X			Learning trip	Correlation	+	+	+	0	0				8	9	180
4	Visscher et al. [74]	2009	USA	*Zoo Biology*	Learning trip	X						X			Lecture/guided tour	Causal relationship	+								10	10	67
5	Nazaruk and Klim-Klimaszewska [66]	2017	Poland	*Journal of Baltic Science Education*	Learning trip				X	X		X			Urban/rural children	Causal relationship	0	NA							6	6	90
6	Kian et al. [18]	2021	Canada	*Frontiers in Psychology*	Camp		X					X			Age	Causal relationship	+							+	4	10	122
7	Jakobsson et al. [73]	2024	Sweden	*International Journal of Science Education*	Learning trip	X				X		X	X	4	Repetition	Causal relationship	0			+	+				10	11	81
8	Conrad et al. [19]	2021	USA	*Journal of Experimental Child Psychology*	Family outing	X				X		X			Anthropomorphisms	Causal relationship	0							+	4	7	48
9	Rahm and Ash [20]	2008	Canada, USA	*Learning Environments Research*	Family outing, Out-of-school		X	X			X	X	X	48	Participation of socially disadvantaged people	Descriptive study					+				9	12	4
10	Collins et al. [28]	2021	Ireland	*Anthrozoos*	Learning trip, Camp			X			X				Enrichment	Causal relationship							+		6	12	1127
11	Collins et al. [59]	2020	Ireland	*Environmental Education Research*	Learning trip, Camp	X				X		X	X	24	Enrichment	Causal relationship	+	+	0						9	12	110
12	Collins et al. [75]	2020	Ireland	*The Journal of Environmental Education*	Learning trip	X		X		X		X			Enrichment	Causal relationship	+	+	0						9	12	501
13	Campana et al. [62]	2023	USA	*Journal of Librarianship and Information Science*	Out-of-school			X			X				Theatre	Descriptive study						+			3	6	121
14	Seybold et al. [68]	2014	Germany	*International Journal of Science and Mathematics Education*	Learning trip	X				X		X	X	7	Combination of school lesson and zoo	Causal relationship	0			-	0				9	13	1013

**Table A3:** Overview of studies found—Part 2. +: positive result, -: negative result, 0: no result, NA: not applicable. If ‘+’, ‘-’, ‘0’ or ‘NA’ appears, this was investigated in the study. If ‘X’ appears, this was used in the study.

No.	Article (Authors)	Year	Country of Authors	Journal	Type of Visit	Instrument				Time of Measurement				Distance Follow-Up (Weeks)	Category of the Examined/Varied Variable	Investigated Relationship	Dependent or Observed Variable								Sample		
						Questionnaire	Interview	Observation	Other	Pre	On-Site	Post	Follow-Up				Knowledge	Behaviour	Attitudes	Motivation	Interest	Learning Behav.	Communication	Other	Age Min	Age Max	Sample Size
15	Sattler and Bogner [76]	2016	Germany	*Environmental Education Research*	Learning trip	X				X		X	X	6	Sation work	Causal relationship	+								15	17	117
16	Sandberg et al. [21]	2011	Netherlands	*Computers & Education*	Learning trip	X				X		X			Inclusion of virtual media	Causal relationship								+	8	10	75
17	Wünschmann et al. [77]	2017	Germany	*Research in Science Education*	Learning trip	X				X		X	X	3	Learning trip	Causal relationship	+			0					8	10	65
18	Randler et al. [78]	2012	Germany	*Journal of Science Education and Technology*	Learning trip	X				X	X	X	X	6	Student-centred approach	Causal relationship	+								10	12	845
19	Douglas and Katz [57]	2009	USA	*Afterschool Matters*	Out-of-school		X	X		X		X			Specific education programme	Causal relationship	+	+	+						10	12	20
20	Collins and O’ Riordan [79]	2022	Ireland	*Environmental Education Research*	Learning trip, Camp	X		X		X	X	X			Enrichment	Causal relationship	+	+	0						6	12	501
21	Chung et al. [45]	2019	USA	*International Journal of Information and Learning Technology*	Family outing	X	X			X		X			Inclusion of virtual media	Causal relationship	+				+			NA	5	12	91
22	Bølling et al. [65]	2017	Denmark	*International Journal of Primary, Elementary and Early Years Education*	Learning trip		X	X			X	X			Learning trip	Descriptive study					+				12	13	26
23	Dohn [54]	2011	Denmark	*Science Education*	Learning trip		X	X	X	X	X	X			Learning trip	Causal relationship					+				17	19	16
24	Basten et al. [80]	2014	Germany	*Science Education*	Learning trip	X				X		X	X	10	Motivation through accompanying persons	Causal relationship	0			+					10	12	206
25	Da Silva et al. [60]	2021	Brazil	*Journal of Ethnobiology and Ethnomedicine*	Learning trip	X				X		X			Direct contact with animals	Causal relationship			+						7	17	72
26	Jensen [29]	2014	UK	*Conservation Biology*	Learning trip	X				X		X			Lecture/guided tour	Causal relationship	+		+	NA					7	15	2839
27	Kleespies et al. [70]	2020	Germany	*Frontiers in Psychology*	Learning trip	X				X		X			Direct contact with animals, conversation with animal keepers	Causal relationship			+						16	19	608

**Table A4:** Overview of studies found—Part 3. +: positive result, -: negative result, 0: no result, NA: not applicable. If ‘+’, ‘-’, ‘0’ or ‘NA’ appears, this was investigated in the study. If ‘X’ appears, this was used in the study.

No.	Article (Authors)	Year	Country of Authors	Journal	Type of Visit	Instrument				Time of Measurement				Distance Follow-Up (Weeks)	Category of the Examined/Varied Variable	Investigated Relationship	Dependent or Observed Variable								Sample		
						Questionnaire	Interview	Observation	Other	Pre	On-Site	Post	Follow-Up				Knowledge	Behaviour	Attitudes	Motivation	Interest	Learning Behav.	Communication	Other	Age Min	Age Max	Sample Size
28	Badger and Shapiro [46]	2019	UK	*Cognitive Development*	Learning trip				X	X		X			Direct contact with animals	Causal relationship								0	5	6,8	252
29	Marth and Bogner [81]	2017	Germany	*Studies in Educational Evaluation*	Learning trip	X				X		X	X	48	Specific education program	Causal relationship	+								10	13	324
30	Davidson et al. [47]	2010	USA, New Zealand, Canada	*Science Education*	Learning trip	X	X	X	X	X	X	X	X	16	Learning trip	Descriptive study	0		0	0	0	0	0	+	11	13	67
31	Counsell et al. [82]	2024	UK	*Environmental Education Research*	Learning trip	X				X		X			Specific education program	Causal relationship	+	+	+						7	11	2099
32	Dohn [55]	2013	Denmark	*International Journal of Science Education*	Learning trip		X	X		X	X	X			Lecture/guided tour	Descriptive study					+				17	19	21
33	Penn [48]	2009	UK	*Zoo Biology*	unclear		X					X			Theatre	Causal relationship	0							+	4	10	348
34	Ocular et al. [83]	2022	USA	*Frontiers in Psychology*	Family outing	X		X			X		X	3	Parent–child conversation	Causal relationship							+/0		3	8	62
35	Collins et al. [84]	2019	Ireland	*Applied Animal Behavior Science*	Learning trip, Camp			X			X				Enrichment	Causal relationship		+							6	12	1127
36	Joy et al. [85]	2021	USA, UK	*Frontiers in Psychology*	Family outing			X			X				Zoo educator	Causal relationship						0			3	14	63
37	Massarani et al. [63]	2022	Brazil	*Science Education*	Family outing		X	X		X	X				Parent–child conversation	Descriptive study			+		+		+		7	12	7
38	Unger and Fisher [64]	2019	USA	*Journal of Experimental Child Psychology*	Camp				X	X		X			Camp	Causal relationship	+								4	9	60
39	Faria and Chaga [56]	2013	Portugal	*International Journal of Science Education*	Learning trip	X		X			X	X			Lecture/guided tour	Descriptive study				+		+			6	18	191
40	Gillespie and Melber [49]	2014	USA	*The Journal of Museum Education*	Learning trip	X	X	X		X	X	X			Specific education program	Causal relationship	+							+	12	14	110
41	Heim and Holt [67]	2022	USA	*Journal of Experiential Education*	Learning trip	X				X		X			Self-directed learning	Causal relationship				0	0	NA			18	22	78

**Table A5:** Overview of studies found—Part 4. +: positive result, -: negative result, 0: no result, NA: not applicable. If ‘+’, ‘-’, ‘0’ or ‘NA’ appears, this was investigated in the study. If ‘X’ appears, this was used in the study.

No.	Article (Authors)	Year	Country of Authors	Journal	Type of Visit	Instrument				Time of Measurement				Distance Follow-Up (Weeks)	Category of the Examined/Varied Variable	Investigated Relationship	Dependent or Observed Variable								Sample		
						Questionnaire	Interview	Observation	Other	Pre	On-Site	Post	Follow-Up				Knowledge	Behaviour	Attitudes	Motivation	Interest	Learning Behav.	Communication	Other	Age Min	Age Max	Sample Size
42	Kruse and Card [86]	2004	USA	*The Journal of Environmental Education*	Camp	X				X		X	X	4	Participation in animal care	Causal relationship	+	+	+						10	18	383
43	Cainey et al. [87]	2012	UK	*Educational Research and Evaluation*	Learning trip		X	X	X	X		X			Lecture/guided tour	Causal relationship	+								4	11	138
44	Buchholz and Pyles [88]	2018	USA	*The Reading Teacher*	Learning trip		X	X	X	X	X	X			Learning trip	Descriptive study	NA	NA	NA	NA	NA	NA	NA	NA	5	6	28
45	Tuttle et al. [89]	2017	USA	*School Science and Mathematics*	Family outing			X			X				Navigators	Descriptive study							+		3	9	101
46	Wheaton and Ash [50]	2008	USA	*Journal of Museum Education*	Camp		X	X			X	X	X	24	Camp	Descriptive study								+	12	13	2
47	Kras [58]	2022	USA	*Community College Journal of Research and Practice*	Learning trip	X						X			Specific education program	Causal relationship	+		+	+					18	22	14
48	Kelly et al. [90]	2022	USA	*The Social Science Journal*	Family outing	X		X			X	X			Parent–child conversation	Correlation							+		3	8	22
49	Kisiel et al. [51]	2012	USA	*Science Education*	Family outing		X	X			X	X			Touch tank	Descriptive study							+	+	3	17	41
50	Kopczak et al. [91]	2015	USA	*Environmental Education Research*	Family outing	X	X	X			X	X			Conservation with animal keepers	Descriptive study							+		3	17	41
51	Tofield et al. [53]	2003	New Zealand	*Research in Science & Technological Education*	Learning trip		X	X		X	X	X			Learning trip	Descriptive study	+		+	+		+			6	17	100
